# Cost-effectiveness of digital therapeutics for essential hypertension

**DOI:** 10.1038/s41440-022-00952-x

**Published:** 2022-06-20

**Authors:** Akihiro Nomura, Tomoyuki Tanigawa, Kazuomi Kario, Ataru Igarashi

**Affiliations:** 1 Innovative Clinical Research Center, Kanazawa University, Kanazawa, Japan; 2 Department of Biomedical Informatics, CureApp Institute, Karuizawa, Japan; 3 CureApp, Inc., Tokyo, Japan; 4 Division of Cardiovascular Medicine, Department of Medicine, Jichi Medical University School of Medicine, Tochigi, Japan; 5 Unit of Public Health and Preventive Medicine, Yokohama City University School of Medicine, Yokohama, Japan; 6 Department of Health Economics & Outcomes Research, Graduate School of Pharmaceutical Sciences, Faculty of Pharmaceutical Sciences, The University of Tokyo, Tokyo, Japan

**Keywords:** Essential hypertension, Digital therapeutics, Cost-effectiveness, Lifestyle modification

## Abstract

Hypertension increases the risk of cardiovascular and other diseases. Lifestyle modification is a significant component of nonpharmacological treatments for hypertension. We previously reported the clinical efficacy of digital therapeutics (DTx) in the HERB-DH1 trial. However, there is still a lack of cost-effectiveness assessments evaluating the impact of prescription DTx. This study aimed to analyze the cost-effectiveness of using prescription DTx in treating hypertension. We developed a monthly cycle Markov model and conducted Monte Carlo simulations using the HERB-DH1 trial data to investigate quality-adjusted life-years (QALYs) and the cost of DTx for hypertension plus guideline-based lifestyle modification consultation treatment as usual (TAU), comparing DTx + TAU and TAU-only groups with a lifetime horizon. The model inputs were obtained from the HERB-DH1 trial, published or publicly available data, and expert assumptions. The incremental cost-effectiveness ratio (ICER) per QALY was used as the benchmark for cost-effectiveness. We performed probabilistic sensitivity analyses (PSAs) using the Monte Carlo simulation with two million sets. The DTx + TAU strategy produced 18.778 QALYs and was associated with ¥3,924,075 ($34,122) expected costs, compared with 18.686 QALYs and ¥3,813,358 ($33,160) generated by the TAU-only strategy over a lifetime horizon, resulting in an ICER of ¥1,199,880 ($10,434)/QALY gained for DTx + TAU. The monthly cost and attrition rate of DTx for hypertension have a significant impact on ICERs. In the PSA, the probability of the DTx arm being a cost-effective option was 87.8% at a threshold value of ¥5 million ($43,478)/QALY gained. In conclusion, the DTx + TAU strategy was more cost-effective than the TAU-only strategy.

## Graphical abstract

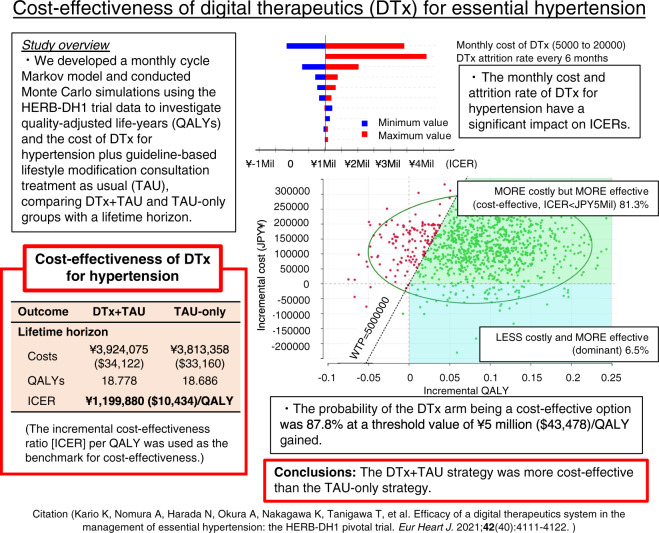

## Introduction

Hypertension or elevated blood pressure (BP) is a serious health condition that significantly increases the risks for cardiovascular diseases (CVDs), heart failure (HF), atrial fibrillation (AFib), renal dysfunction, and other disorders [1]. Hypertension affects 1.28 billion people and is a leading modifiable risk factor for mortality worldwide [1, 2]. In Japan, there are more than 43 million patients with hypertension, with one of the highest prevalence rates among the Organization for Economic Co-operation and Development countries [3]. In total, 50% of all CVD deaths, 59% of coronary artery disease (CAD) deaths, and 52% of stroke deaths are attributed to hypertension [4]. Additionally, the total medical cost of these CVDs has reached nearly $100 billion in Japan [5]. Therefore, managing hypertension is one of the most critical factors in preventing CVDs and reducing medical costs in Japan. For this purpose, Health Japan 21 (the second edition) aims to reduce the average systolic BP (SBP) of the Japanese population by 4 mmHg within 10 years by raising awareness and promoting lifestyle modification including consuming a healthy diet, increasing physical activity, and reducing alcohol consumption at the national level [6].

Lifestyle modification is a major component of nonpharmacological treatment for hypertension and is as essential as antihypertensive pharmacological treatment [7]. In principle, hypertensive patients at low risk for CVD should first modify their lifestyle, and antihypertensive drug therapy should be initiated at an appropriate time according to the patients’ future CVD risks. Lifestyle modification plays a vital role in lowering blood pressure because of its antihypertensive and synergistic effects along with antihypertensive drugs [8, 9]. However, the physician’s limited outpatient time is insufficient to provide guideline-based hypertension management, including lifestyle modification suited for each patient’s diverse lifestyle [10]. As a result, lifestyle modification has not achieved its full effect in reducing BP.

To maximize the potential treatment effect of lifestyle modification for hypertension, we developed a prescription digital therapeutics (DTx) for hypertension, the HERB system [11]. The HERB system consists of a HERB Mobile smartphone app for patients and web-based patient management software for primary doctors [11]. We previously reported the HERB-DH1 phase III randomized controlled trial (RCT) results of DTx for essential hypertension. In this trial, we randomly assigned 390 essential hypertension patients aged ≤65 years who were not taking antihypertensive medication to the DTx intervention group or the control group. The results showed that the primary outcome of the mean reduction in 24-h SBP from baseline to 12 weeks was significantly larger in the DTx intervention group than in the control group [12].

Although the HERB-DH1 trial demonstrated the clinical efficacy of DTx, there is still a lack of cost-effectiveness assessments evaluating the impact of the prescription DTx for hypertension on health care costs along with clinical efficacy. The cost-effectiveness of DTx for opioid use disorder or that for low back pain was recently evaluated using RCT results [13–15]. However, no study has assessed the cost-effectiveness of prescription DTx for hypertension using RCT data. Exploring the appropriate DTx pricing determined by cost-effectiveness analysis is crucial in countries including Japan, whose DTx costs could be reimbursed by public health insurance.

Here, we aimed to analyze the cost-effectiveness of using prescription DTx in treating hypertension using results from the HERB-DH1 phase III RCT.

## Methods

We developed an economic model to assess the cost and effectiveness of DTx for essential hypertension. The model investigated quality-adjusted life-years (QALYs) and the cost of DTx for hypertension plus guideline-based lifestyle modification consultation treatment as usual (TAU) (DTx + TAU) vs. TAU only with a lifetime horizon and a 1-month cycle length. We constructed the model from the perspective of a public healthcare payer. We compared the cost-effectiveness of DTx + TAU with TAU alone for the treatment of CAD, stroke, HF, and AFib. We used the parameters of the HERB-DH1 phase III clinical trial [12] as inputs. The HERB-DH1 trial is an open-label RCT of HERB prescription DTx in 390 patients with essential hypertension. At 12 weeks, the DTx + TAU intervention group exhibited a significant reduction in SBP compared with the TAU-only control group (−10.6 mmHg in the DTx + TAU group vs. −6.2 mmHg in the TAU-only control group by a home BP measurement). The present study evaluated outcomes as the incremental cost-effectiveness ratio (ICER) per QALY gained. The model inputs were obtained from the HERB-DH1 trial data, published academic papers, publicly available data, and expert assumptions. We conducted the analyses according to the Consolidated Health Economic Evaluation Reporting Standards (CHEERS) statement and the Ethics Guidelines for Medical and Biological Research Involving Human Subjects in Japan. The authors have the right to publish, regardless of the outcome. The Institutional Review Board of Kanazawa University, Japan approved the study protocol.

### Model structure and clinical pathway

**Fig. 1:**
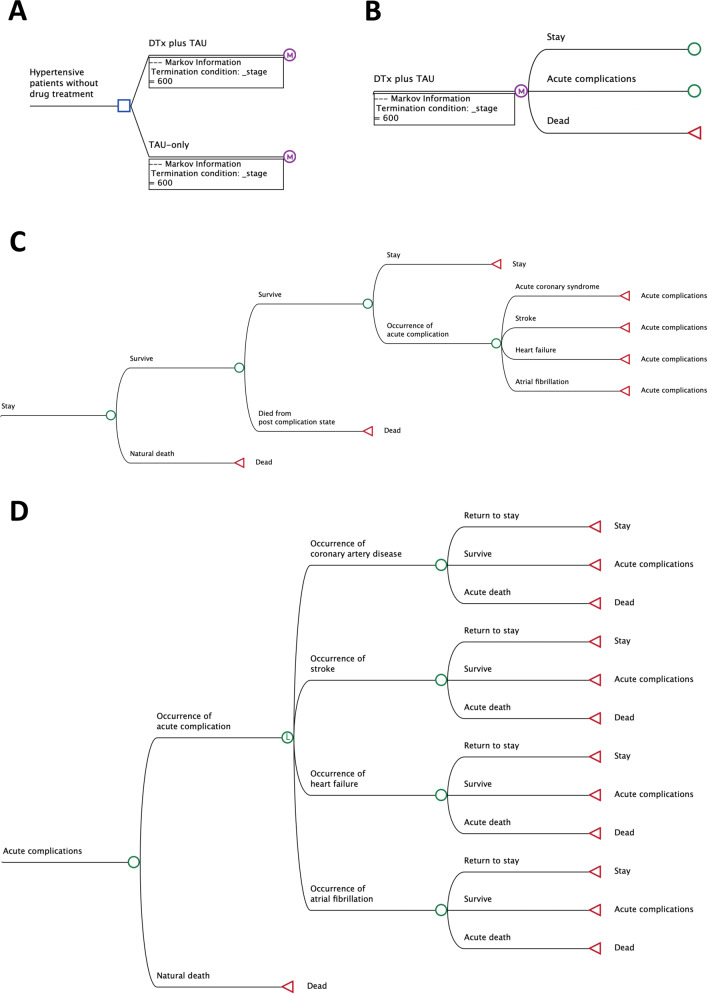
Structure of the simulation model. **A** Decision tree. **B** Structure of the Markov model. **C** Structure of the Stay (no acute complications) module. **D** Structure of the acute complications module. TreeAge Pro Healthcare software was used to create this simulation model. The blue square indicates the decision node; M in the purple circle indicates the Markov node; the green circle indicates the chance node; L in the green circle indicates the logic node; and the red triangle indicates the terminal node. The terminal condition of _stage = 600 denotes that the maximum cycle length would be 600 times. Given that one cycle is equal to one month, 600 iterations means that the time horizon was set to 50 years (600 months), or a lifetime. We set three health statuses: stay (acute complication-free status), acute complication, or death. The occurrence of acute complications or death rates varies according to age, home systolic blood pressure level, and the latest acute complication history. Additionally, acute complication status includes four hypertension-related complications: coronary artery disease, stroke, heart failure, and atrial fibrillation

We developed a monthly cycle Markov model and conducted Monte Carlo simulations to estimate the efficacy of DTx + TAU (Fig. 1). A Markov model is a stochastic process that transitions from one state to another, can be implemented from robust to model medical environments and addresses problems affecting stochastic and sequential decisions over time [16]. The limitation of the pure Markov model was its memoryless property; the model cannot distinguish between those who never experienced any complication and those who did and recovered (surviving in the chronic phase). Under the Monte Carlo simulation, each simulated patient enters the Markov model, allowing one’s entire movement to be captured. In this model, we can assign different costs and QALYs for those with/without experience of specific complications, even if they remained in the same health state. Moreover, using Monte Carlo simulation, we could individually simulate patient characteristics, while only the average value could be used in the pure Markov model. In this study, a Monte Carlo simulation, with 1,000,000 iterations, was performed to reflect the variation in patient characteristics, such as age and treatment effects, mainly derived from those of enrollees for the clinical trial. The model consisted of patients who received DTx + TAU and those who received TAU only. We modeled three different health statuses as follows: “stay” (healthy without any acute complications), “acute complications*”* (any acute complications of hypertension), and “death.” Acute complications included CAD, stroke, HF, and AFib. We also considered natural deaths in the model. Hypertensive patients who avoid natural death can stay in the same acute complication-free status (“stay”) or experience acute complications of CAD, stroke, HF, or AFib. In this model, a patient with one of the acute complications must maintain the “acute complication” status or die from the complication within 12 months, then recover and switch from the “acute complication” status to the “stay” status with a postcomplication (chronic) tag [return to the “stay” status but had slightly higher mortality rates than natural deaths in patients after having either CAD, stroke, or HF]. We did not consider a patient with more than one complication within 12 months of the latest occurrence of acute complications.

### Time horizon

We set lifetime years (50 years) as the time horizon for our study. We also conducted scenario analyses to shift the time horizon to 20, 30, and 40 years.

### Model population and intervention

The study population was derived from the HERB-DH1 trial. The participants were grade I or II hypertensive patients without antihypertensive drug treatment, with a mean age of 52 years; 20% were female, 16% were current smokers, and the proportions of dyslipidemia and diabetes were 50 and 7%, respectively. The average SBP was 145 mmHg for 24-h SBP according to ambulatory BP monitoring and 148 mmHg for morning home SBP according to home BP monitoring. This model used log-normalized baseline home SBP distribution from HERB-DH1 trial data (Table 1). Additionally, we modeled the annual SBP increase in both groups by 0.5 mmHg from the national survey [7].

**Table 1:** Model inputs

Data	Input	Low^a^	High^a^	Source
Age	52.3 years	–	–	Kario [12, 40]
Baseline SBP	148 mmHg	–	–	Kario [12, 40]
Annual BP increase	0.5 mmHg	0.39	0.65	Umemura [7]
Annual mortality rates by complications				
Coronary artery disease				
First 1 month after event onset	5.0%/month	0	5.0	Komiyama [19]
>1 month (post-CAD)	0.98%	0.93	1.74	Goto [20]
Stroke				
First 1 month after event onset	12.9% / month	9.7	16.1	Takashima [23]
>1 month (post-Stroke)	1.29%	–	–	Goto [20]
Heart failure				
First 12 months after event onset	11.5%	10.1	13.0	Shiraishi [21]
>13 months (post-HF)	2.5%	–	–	Tsuji [22]
Atrial fibrillation				
First 12 months after event onset	1.83%	1.28	2.56	Goto [20]
>13 months (post-AFib)	Same as the natural mortality rate			–
Annual incidence rates of each complication among hypertension patients				
Coronary artery disease	0.460%	0.345	0.575	Kaneko [18], Turin [24]
Stroke	1.592%	1.194	1.990	Kaneko [18], Turin [25]
Heart failure	0.889%	–	–	Kaneko [18], Fujimoto [26]
Atrial fibrillation	0.232%	–	–	Kaneko [18]
Annual costs				
DTx for hypertension	¥120,000 ($1,043)	60,000	240,000	Assumption
Acute phase (First 12 months after event onset)				
Coronary artery disease	¥2,156,290 ($18,750)	1,617,218	2,695,362	Kamae [27]
Stroke	¥1,440,107 ($12,523)	1,080,080	1,800,134	Kamae [27]
Heart failure	¥770,428 ($6,699)	577,821	963,035	Mizuno [28]
Atrial fibrillation	¥120,000 ($1,043)	90,000	150,000	Kamae [29]
Post-complication phase (>12 months)				
Coronary artery disease	¥495,600 ($4,310)	459,996	570,000	Kodera [30]
Stroke	¥318,387 ($2,769)	262,992	372,996	Kodera [30]
Heart failure	¥770,428 ($6,699)	577,821	963,035	Mizuno [28]
Atrial fibrillation	¥120,000 ($1,043)	90,000	150,000	Kamae [29]
Health utilities				
Normal	0.950	0.760	1.000	–
Coronary artery disease (increment)	−0.180	−0.135	−0.225	Kodera [30]
Stroke (increment)	−0.157	−0.117	−0.196	Hattori [32]
Heart failure (increment)	−0.101	−0.076	−0.126	Clarke [33]
Atrial fibrillation (increment)	−0.000	–	–	Assumption
Death (increment)	−0.950	–	–	–
Discount rates	2%	0	4	Shiroiwa [31]

*AF* atrial fibrillation, *BP* blood pressure, *CAD* coronary artery disease, *CI* confidence interval, *HF* heart failure, *SBP* systolic blood pressure

^a^Range for sensitivity analysis

The DTx + TAU group received the prescription DTx, HERB Mobile system, and lifestyle modifications to manage hypertension as recommended by the Japanese Society of Hypertension (JSH) guidelines [7]. The HERB Mobile system includes the HERB Mobile smartphone app and a web-based management application for health care providers. The smartphone app retrieves each patient’s baseline input data, and daily home BP measurements are transferred to the cloud server and assessed to plan a personalized lifestyle modification program to lower BP. In addition, primary physicians can simultaneously browse their patients’ data, such as BP measurements, daily activities, or progress in the proposed program, using a web-based application [11, 12]. The TAU-only group was also encouraged to make lifestyle modifications for hypertension based on the JSH guidelines by their primary physicians or themselves. We assumed that the hypertensive patients in the DTx + TAU group would have an average home SBP reduction of 10.6 mmHg, while those in the TAU-only group would have a 6.2 mmHg reduction according to a previous study [12]. In this simulation model, we used DTx + TAU and TAU-only effect distributions derived from the HERB-DH1 trial data (Supplementary Fig.). Since DTx adherence could decrease with time, we set the model such that 25% of the remaining users in the DTx + TAU group discontinued use of the DTx every 6 months.

### Mortality and complications

We obtained the annual natural death rates from the Japan Vital Statistics for 2022 [17]. The annual mortality and occurrence rates of each complication are summarized in Table 1. We set both acute and poststate (chronic) mortality rates for each complication [18–23].

The occurrence rates of each complication were adjusted according to BP categories (Supplementary Table) [18]. Additionally, the annual incidence rates of CAD and stroke were calibrated according to the previously reported lifetime risks [24, 25]. In addition, the annual incidence rate of HF was calibrated according to patient age (hazard ratios [HRs] of 2.5, 5.0, and 10 for people in their 60 s, 70 s, and ≥80 s) [26]. Categories of normal BP, elevated BP, grade I HT, and grade II HT were defined as home SBPs of <125, 125–134, 135–144, and ≥145 mmHg, respectively [7]. Since the cycle length of the model was 1 month, we converted the annual rates to monthly rates to input into the model.

### Costs

A list of the cost data is presented in Table 1. We performed an economic estimation from Japan’s public health care payer perspective, under which only medical costs were considered. The costs consist of treatment and hospitalization expenses but not transportation fees or family care service costs. We set the monthly cost of HERB DTx at ¥10,000 ($86.96 US: $1 US = ¥115 as of January 2022), including app purchases and subscription fees. The cost of each complication (CAD, stroke, HF, and AFib) was also derived from previous studies [27–30]. We set an annual cost reduction of 2% according to the Japanese guideline for cost-effectiveness analyses [31].

### Health-related quality of life and utilities

We derived the utility values of CAD, stroke, HF, and AFib from published studies based on the European Quality of Life-5 Dimensions Questionnaire (Table 1) [30, 32, 33]. We calculated QALYs by multiplying the time duration in a specific health state by the utility value associated with the state. Both cost and outcome were discounted at a 2% discount rate [31].

### Main analysis

We calculated the ICER between DTx + TAU and TAU-only strategies over the lifetime horizon using the estimated model parameters and cost and effectiveness assumptions.

### Sensitivity and scenario analyses

We tested the impact of the uncertainties around the model parameters on the overall cost-effectiveness results using one-way deterministic and probabilistic sensitivity analyses. First, we performed one-way deterministic sensitivity analyses by changing the treatment effect, cost, and utility parameters over each range between low and high values (Table 1 and Supplementary Table). Then, we conducted probabilistic sensitivity analyses to evaluate the sensitivity of the results to simultaneous variable changes using a set of two million (2000 iterations × 1000 individuals) simulated results with specific probability distributions of the input parameters. We assumed a Weibull distribution for age; log-normal distribution for initial SBP input; normal distributions for HRs of each complication according to BP grade, mortality rates, and health utilities; beta distributions for clinical event incidence rates; and gamma distributions for costs. In addition, we drew the probability of the cost-effectiveness acceptability curve to evaluate the cost-effectiveness probability at a willingness-to-pay threshold of ¥5 million ($43,478) [34]. Additionally, we conducted two scenario analyses as follows: (#1) simulating different time horizons of 20, 30, and 40 years; and (#2) setting an alternative DTx app attrition rate of 10% every six months. All model analyses were conducted using TreeAge Pro HealthCare version 2022 R1.0 (TreeAge Software, LLC, MA, USA).

## Results

### Main analysis

The model predicted that the DTx + TAU strategy produced 18.778 QALYs and was associated with ¥3,924,075 ($34,122) expected costs compared to 18.686 QALYs and ¥3,813,358 ($33,160) generated by TAU-only treatment over a lifetime horizon. The introduction of DTx would increase costs by ¥110,717 ($962) and prolong QALYs by 0.092. The ICER of DTx + TAU against TAU would be ¥1,199,880 ($10,434)/QALY gained, which was well below the hypothetical threshold value in Japan of ¥5 million/QALY gained (Table 2).

**Table 2:** Cost-effectiveness of DTx for hypertension

Outcome	DTx + TAU	TAU-only	Changes
Lifetime horizon			
Costs	¥3,924,075 ($34,122)	¥3,813,358 ($33,160)	¥110,717 ($962)
QALYs	18.778	18.686	0.092
ICER	¥1,199,880 ($10,434)/QALY^a^		
40-year time horizon			
Costs	¥3,705,428	¥3,591,385	¥114,043
QALYs	18.483	18.395	0.089
ICER	¥1,284,159 ($11,167)/QALY		
30-year time horizon			
Costs	¥2,996,162	¥2,869,894	¥126,268
QALYs	17.195	17.124	0.071
ICER	¥1,788,344 ($15,551)/QALY		
20-year time horizon			
Costs	¥1,820,066	¥1,664,483	¥155,583
QALYs	13.939	13.903	0.037
ICER	¥4,235,677 ($36,832)/QALY		

*DTx* digital therapeutics, *ICER* incremental cost-effectiveness ratio, *QALY* quality-adjusted life year, *TAU* treatment as usual

^a^The ICER was calculated by the following numbers before rounding (the cost change: 110716.867722453 yen; and the QALY change: 0.092273268384691)

### Sensitivity analyses

**Fig. 2:**
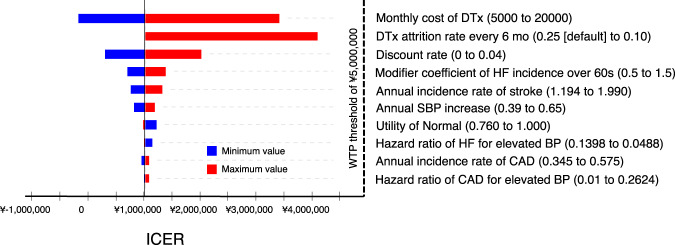
One-way sensitivity analysis results. Bar plots indicate the changes in ICER values in each parameter range (minimum value in blue and maximum value in red). The monthly cost of DTx had the largest impact on the ICER value, followed by the DTx attrition and discount rates. Even the highest ICER value observed in this diagram was below the threshold of ¥5 million/QALY gained. Abbreviations: BP blood pressure, CAD coronary artery disease, DTx digital therapeutics, HF heart failure, ICER incremental cost-effectiveness ratio, WTP willingness-to-pay

We conducted one-way deterministic and probabilistic sensitivity analyses. The results of the one-way deterministic sensitivity analyses with the tornado diagram are shown in Fig. 2. The monthly cost of DTx, which ranged from ¥5,000 ($43.48)/month to ¥20,000 ($173.91)/month, had the largest impact on the ICER value, followed by the DTx attrition and discount rates. According to the one-way threshold analysis for the monthly DTx cost, the DTx + TAU arm became the dominant strategy when the cost was below ¥5,163 ($44.90) per month. Even the highest ICER value observed in this diagram was still below the threshold value of ¥5 million/QALY gained.

**Fig. 3:**
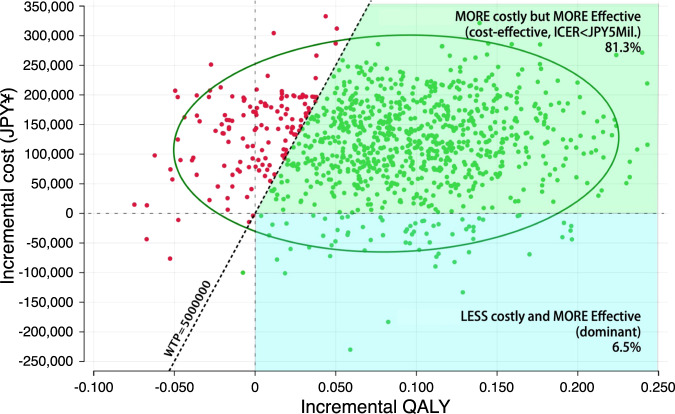
Cost-effectiveness plot. Each dot represents one Monte Carlo simulation result of incremental QALY and cost. The green plots indicate that the simulation outputs demonstrated that DTx + TAU was cost-effective in terms of ICER less than ¥5 million/QALY gained, whereas the red plots denote that the outputs did not show that DTx + TAU was cost-effective or that ICER exceeded the threshold value. The green ellipse indicates the 95% confidence area of the simulations. The probability rates of dominance (the area filled with light blue) and cost-effectiveness (the area filled with light green) were 6.5% and 81.3%, respectively. Abbreviations: ICER incremental cost-effectiveness ratio, JPY Japanese yen, QALY quality-adjusted life years, WTP willingness-to-pay

**Fig. 4:**
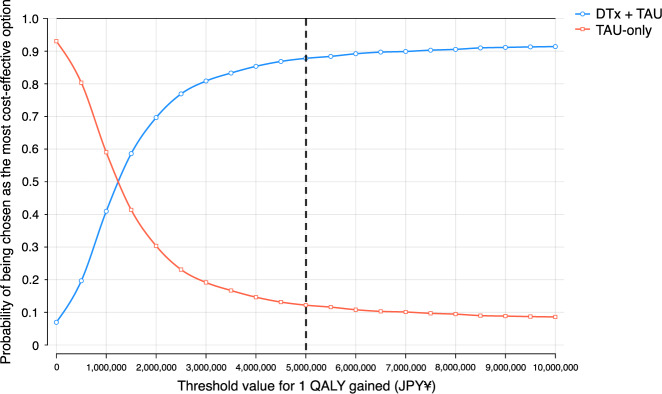
Cost-effectiveness acceptability curve in the DTx + TAU and TAU-only groups. The *X*-axis indicates the threshold value for 1 QALY gained (we preset the threshold value as ¥5 million/QALY gained, shown as the dashed line), and the *Y*-axis denotes the probability of being chosen as the most cost-effective option. As the threshold value increases, the probability of choosing the DTx + TAU strategy (the blue curve) sharply increases, whereas that of choosing the TAU-only strategy (the red curve) decreases. At the intersection of the two curves, the threshold value was approximately ¥1,200,000/QALY gained, which was sufficiently below the preset threshold of ¥5 million/QALY gained. Abbreviations: DTx digital therapeutics, JPY Japanese yen, QALY quality-adjusted life years, TAU treatment as usual

The results of the probabilistic sensitivity analysis are shown with incremental cost-effectiveness scatter plots (Fig. 3) and a cost-effectiveness acceptability curve (Fig. 4). According to these figures, the probability of dominance (DTx less costly and more effective) and cost-effectiveness (DTx more costly and more effective, the ICER was less than ¥5 million/QALY gained) were 6.5% and 81.3%, respectively.

### Scenario analyses

In scenario #1, by changing the simulation duration, the ICERs for the DTx + TAU treatment were inversely proportional to the extent of the time horizon (Table 2). In scenario #2, using an alternative DTx attrition rate of 10% every six months, ICER was reached in ¥4,330,295 ($37,655)/QALY.

## Discussion

In this study, we assessed the cost-effectiveness of prescription DTx for essential hypertension, in addition to TAU, compared to the TAU-only strategy. We found that the ICER of the DTx + TAU strategy was ¥1,199,880 ($10,434)/QALY against the TAU-only strategy in the lifetime horizon, which was well below the threshold value of ¥5 million/QALY. Moreover, the probability of the cost being below the threshold value was 87.8% in the probabilistic sensitivity analysis. This model analysis demonstrated that DTx + TAU is cost-effective for patients with hypertension.

Several conclusions can be drawn from this study. First, this is the first study to evaluate the cost-effectiveness of DTx for patients with hypertension using the results of an RCT. Nordyke et al. reported the cost-effectiveness of DTx for hypertension with person-to-person health coaching according to data from a retrospective study over a 3-year time horizon [35, 36]. Other than hypertension, DTx for opioid use disorder and that for low back pain also revealed cost-effectiveness in a relatively short time horizon (12 weeks to 3 years) without considering mortality rates [13–15]. Although the time to half DTx usage rate was approximately 15 months in this model, the benefit of the BP-lowering effect persisted for more than three years, resulting in prevention of the trajectory of CVD from hypertension, left ventricular hypertrophy, and atherosclerotic events to HF and CVD deaths. Therefore, a long-term cost-effectiveness analysis would be valuable when considering the practical effects of DTx for chronic diseases such as hypertension.

The monthly cost of DTx for hypertension had the greatest impact on ICERs in the one-way deterministic sensitivity analysis. Lewkowicz et al. pointed out a similar conclusion by evaluating the cost-effectiveness of DTx for low back pain [15]. At a DTx cost of ¥5,000/month, DTx dominated conventional therapy or was a less costly and more effective option. Even when we set the cost at ¥20,000/month, the ICER was approximately ¥3.5 million/QALY, which is still under the threshold value of ¥5 million/QALY (Fig. 2). Recently, sacubitril/valsartan (angiotensin receptor-neprilysin inhibitor), a novel antihypertensive drug, was approved for hypertension management in Japan [37], with a medication cost of ¥6,057 ($52.67) to ¥12,114 ($105.34)/month. Thus, the DTx cost we set to approximately ¥10,000/month as the default might be a reasonable value according to the current simulation model.

The attrition rate of the DTx program is a crucial factor for cost-effectiveness. A smartphone mHealth app usage rate attenuated over time, reporting that approximately 40% of attrition occurred from 6 months to 1 year [38]. Previous DTx studies used for cost-effectiveness analyses also used similar attrition rates [15, 35]. Thus, it could be reasonable to set the attrition rate to 25% for every 6 months in this model, which was approximately 15 months as time to half usage rate since the initiation of DTx. In another scenario, when we changed the rate to 10% (lower than the predefined 25%), the time to half exceeded approximately 40 months, and the ICER was increased to approximately ¥4.3 million/QALY in a lifetime horizon, which was close to the threshold value (Fig. 2). Lewkowicz et al. previously reported that lowering the DTx app attrition rate was essential for cost-effectiveness in a relatively short-term (3-year) horizon [15]. Although the model inputs and target disease were different, it might be possible that high attrition rates along with DTx actual usage duration could substantially impact DTx cost-effectiveness. Achieving good cost-effectiveness for DTx might require sensitive handling to balance the appropriate DTx app usage duration with DTx costs and expected attrition rate.

The degree of the gained ICER using DTx hypertension management surely depends on the degree of BP reduction by quality of the DTx app algorithm, the impact of BP reduction, and the cost of cardiovascular events in each country. A significant difference in home BP reduction (−4.3 mmHg of morning home SBP) was clearly found between the DTx + TAU and TAU-only groups from the previous phase III clinical trial. Notably, this TAU-only group followed the ideal guideline-driven management of hypertension, which used home BP monitoring [7]. Compared with office BP, home BP is more closely associated with cardiovascular event risk [39, 40]. In addition, in Asian countries, the benefit of BP reduction is greater than that in Western countries, especially for stroke [41]. The comparison of cost-effectiveness studies using different DTx apps in different contigs is also needed in the future.

The strength of this study is that, to our knowledge, this is the first study to evaluate the cost-effectiveness of DTx for patients with hypertension using the results from an RCT. Furthermore, we demonstrated that DTx + TAU is cost-effective for patients with hypertension. This study has some limitations. First, we used middle-aged, digital-friendly grade I or II hypertensive patients who were not taking antihypertensive medication for the simulations according to previous RCT results. Thus, caution should be taken when applying our results to a broader population. Second, we modeled the DTx effect as permanent, although the effect estimates were derived from the 6-month duration of the HERB-DH1 trial results. Thus, it might be possible that the treatment effect could attenuate or diminish over time, especially six months after the intervention. Although we set the BP reduction effects even for the TAU-only strategy and age-related annual SBP increases in both groups, this could lower the mortality and complication occurrence rates in the DTx + TAU strategy, resulting in a higher ICER than we concluded in this study. Third, we did not estimate the additive effects of having multiple comorbidities in this model, which could impact lifetime ICER. Fourth, we could not strictly assess the DTx cost-effectiveness in addition to antihypertensive medications since the model patients derived from previous trials were all antihypertensive drug-naïve. Finally, we did not consider reusing DTx after finishing the initial DTx usage according to the attrition rate, which could account for additional costs for the DTx strategy.

In conclusion, DTx + TAU demonstrated good cost-effectiveness in hypertensive patients compared to the TAU-only strategy. DTx cost and its attrition rate largely influence cost-effectiveness, and we need to explore the balance among the DTx application cost, app attrition rate, and effectiveness for target hypertensive patients in clinical practice. DTx is generally reimbursed by Japan’s national universal health insurance system. Thus, it is becoming increasingly essential to evaluate the cost-effectiveness of DTx, as was done in this study, to consider the appropriate value-based reimbursement pricing.

## Supplementary information

SUPPLEMENTAL MATERIAL

Supplementary File for Review
