# Supplementary material for: Cost-effectiveness of digital therapeutics for essential hypertension
Source: Hypertens Res. 2022 Jun 20;45(10):1538–48. doi: 10.1038/s41440-022-00952-x (PMC9474296; doi:10.1038/s41440-022-00952-x)
Supplement: Supplementary file 1 — SUPPLEMENTAL MATERIAL [file 41440_2022_952_MOESM1_ESM.pdf]

Cost-effectiveness of digital therapeutics for essential hypertension

SUPPLEMENTAL MATERIAL

Akihiro Nomura, Tomoyuki Tanigawa, Kazuomi Kario, Ataru Igarashi

Supplemental Table. Hazard ratios of event occurrence for each complication by BP grades.

Upper table represents the list of adjustment factors for acute complication occurrence rates by each BP grades. Lower table shows the range for sensitivity analysis for elevated BP values. We only used the elevated BP range values for the sensitivity analysis.

Abbreviations: BP, blood pressure; HT, hypertension.

Data input	Normal BP	Elevated BP	Grade I HT	Grade II HT	Source
Acute coronary syndrome	1 (ref)	1.14	1.46	1.96	Kaneko, 2021.[1]
Stroke	1 (ref)	1.13	1.35	2.14	Kaneko, 2021.[1]
Heart failure	1 (ref)	1.1	1.3	2.05	Kaneko, 2021.[1]
Atrial fibrillation	1 (ref)	1.07	1.21	1.52	Kaneko, 2021.[1]

Data input	Elevated BP	Low*	High*
Acute coronary syndrome	1.14	1.01	1.30
Stroke	1.13	1.06	1.21
Heart failure	1.1	1.05	1.15
Atrial fibrillation	1.07	0.99	1.17

Supplemental Figure. Distribution of treatment effects for SBP obtained from the HERB-DH1 pivotal trial.[2] A: DTx + TAU group. B: TAU-only group.

Each bar graph represents treatment effects (mmHg) for home morning SBP observed in the HERB-DH1 trial.

Abbreviations: DTx, digital therapeutics; SBP, systolic blood pressure; TAU, treatment as usual.

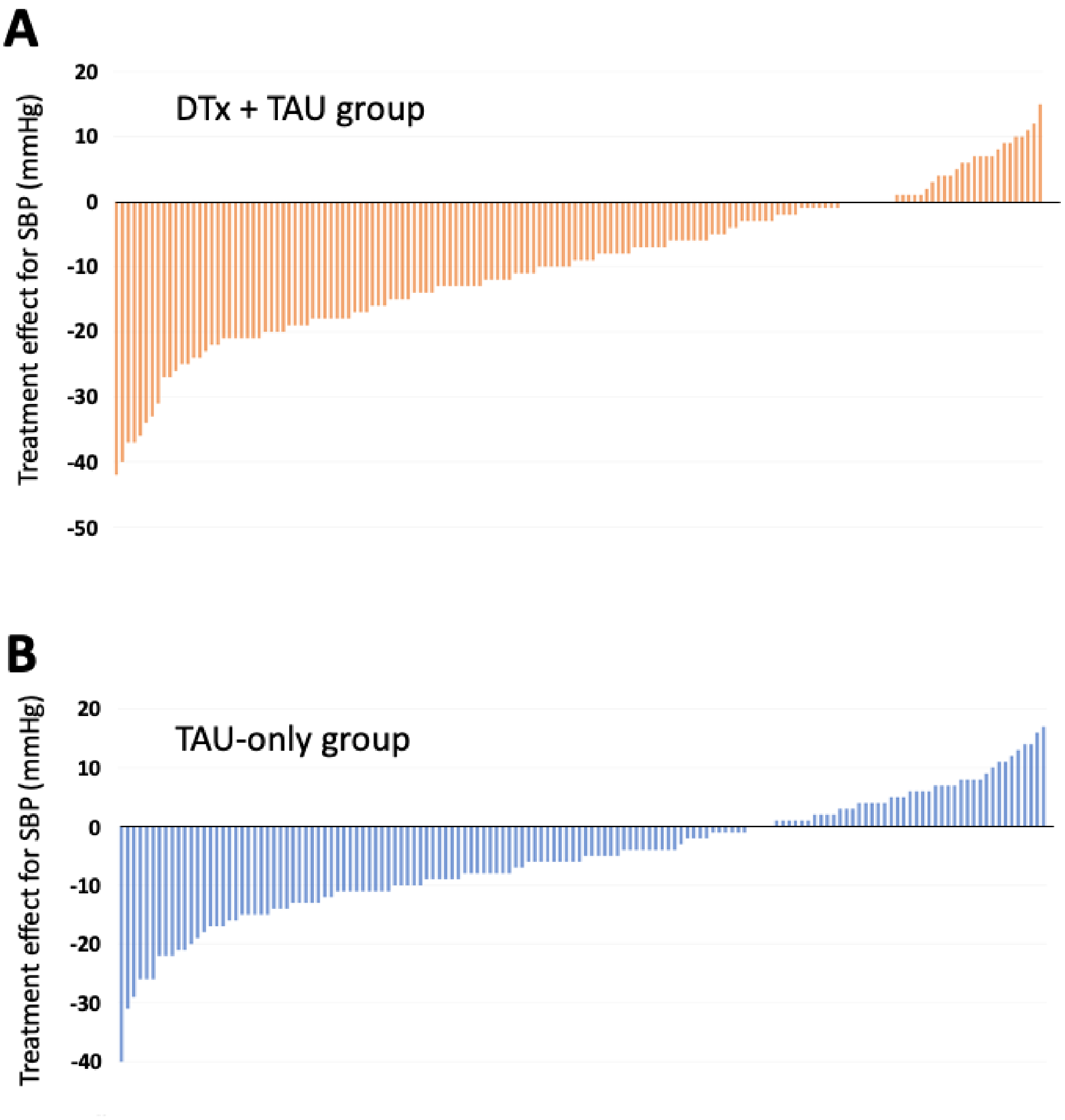

References

1. Kaneko H, Yano Y, Itoh H, Morita K, Kiriyama H, Kamon T, et al. Association of Blood Pressure Classification Using the 2017 American College of Cardiology/American Heart Association Blood Pressure Guideline With Risk of Heart Failure and Atrial Fibrillation. *Circulation.* 2021;**143**(23):2244-2253.
2. Kario K, Nomura A, Harada N, Okura A, Nakagawa K, Tanigawa T, et al. Efficacy of a digital therapeutics system in the management of essential hypertension: the HERB-DH1 pivotal trial. *Eur Heart J.* 2021;**42**(40):4111-4122.
